# Machine Learning for Early Detection of Cognitive Decline in Parkinson’s Disease Using Multimodal Biomarker and Clinical Data

**DOI:** 10.3390/biomedicines12122758

**Published:** 2024-12-03

**Authors:** Raziyeh Mohammadi, Samuel Y. E. Ng, Jayne Y. Tan, Adeline S. L. Ng, Xiao Deng, Xinyi Choi, Dede L. Heng, Shermyn Neo, Zheyu Xu, Kay-Yaw Tay, Wing-Lok Au, Eng-King Tan, Louis C. S. Tan, Ewout W. Steyerberg, William Greene, Seyed Ehsan Saffari

**Affiliations:** 1 Duke-NUS Medical School, National University of Singapore, Singapore 169857, Singapore; raziyeh.mohammadi@duke-nus.edu.sg (R.M.); adeline.ng.s.l@singhealth.com.sg (A.S.L.N.); tay.kay.yaw@singhealth.com.sg (K.-Y.T.); au.wing.lok@singhealth.com.sg (W.-L.A.); louis.tan.c.s@singhealth.com.sg (L.C.S.T.); 2 Department of Research, National Neuroscience Institute, Singapore 308433, Singapore; samuel.ng.y.e@singhealth.com.sg (S.Y.E.N.); choi.xinyi@singhealth.com.sg (X.C.); dede.liana.heng@singhealth.com.sg (D.L.H.); tan.eng.king@singhealth.com.sg (E.-K.T.); 3 Department of Neurology, National Neuroscience Institute, Singapore 308433, Singapore; jayne.tan.yi@singhealth.com.sg (J.Y.T.); deng.xiao@sgh.com.sg (X.D.); shermyn.neo.x.m@singhealth.com.sg (S.N.); xu.zhe.yu@singhealth.com.sg (Z.X.); 4 Department of Biomedical Data Sciences, Leiden University Medical Center, 2333 ZD Leiden, The Netherlands; e.w.steyerberg@lumc.nl; 5 Department of Econometrics, Stern School of Business, New York University, New York, NY 10012, USA; wgreene@stern.nyu.edu

**Keywords:** neurodegenerative disease, cognitive dysfunction, Parkinson’s disease, early diagnosis, risk assessment, machine learning, blood biomarkers, Montreal cognitive assessment, nonmotor symptoms, predictive models

## Abstract

**Background**: Parkinson’s disease (PD) is the second most common neurodegenerative disease, primarily affecting the middle-aged to elderly population. Among its nonmotor symptoms, cognitive decline (CD) is a precursor to dementia and represents a critical target for early risk assessment and diagnosis. Accurate CD prediction is crucial for timely intervention and tailored management of at-risk patients. This study used machine learning (ML) techniques to predict the CD risk over five-year in early-stage PD. **Methods**: Data from the Early Parkinson’s Disease Longitudinal Singapore (2014 to 2018) was used to predict CD defined as a one-unit annual decrease or a one-unit decline in Montreal Cognitive Assessment over two consecutive years. Four ML methods—AutoScore, Random Forest, K-Nearest Neighbors and Neural Network—were applied using baseline demographics, clinical assessments and blood biomarkers. **Results**: Variable selection identified key predictors of CD, including education year, diastolic lying blood pressure, diastolic standing blood pressure, systolic lying blood pressure, Hoehn and Yahr scale, body mass index, phosphorylated tau at threonine 181, total tau, Neurofilament light chain and suppression of tumorigenicity 2. Random Forest was the most effective, achieving an AUC of 0.93 (95% CI: 0.89, 0.97), using 10-fold cross-validation. **Conclusions**: Here, we demonstrate that ML-based models can identify early-stage PD patients at high risk for CD, supporting targeted interventions and improved PD management.

## 1. Introduction

Parkinson’s disease (PD) is a prevalent neurodegenerative disorder impacting millions worldwide, characterized by the gradual deterioration of both motor and non-motor functions [1,2,3]. While motor symptoms such as bradykinesia, resting tremor, rigidity, and postural instability are typically the earliest manifestations, PD is increasingly recognized for its extensive non-motor symptoms, including cognitive dysfunction, autonomic disturbances, and psychiatric issues [4]. Among these, cognitive decline is particularly concerning, as it is associated with a significant risk of progression to dementia, with nearly half of PD patients developing dementia within a decade of diagnosis [5,6,7,8]. Cognitive decline often begins early in the disease course and is accompanied by structural brain changes, including gray matter alterations in the temporal regions, hippocampus, frontal, and parietal lobes, as well as white matter changes in the corpus callosum and cingulate gyrus [9,10]. Recent studies have highlighted the prevalence of mild cognitive impairment in PD (PD-MCI), which affects 20–33% of patients at diagnosis and serves as a precursor to Parkinson’s disease dementia (PDD) [11,12]. Notably, 60–80% of those with PD-MCI progress to PDD within 12 years [13,14]. Furthermore, approximately 30–35% of individuals with early-stage PD experience cognitive decline, emphasizing the need for early detection and intervention [15,16].

Given the profound impact of cognitive decline on PD patients and the significant economic burden it imposes, developing an accurate and cost-effective predictive model for cognitive decline in early PD stages is crucial [17]. Early identification of cognitive decline offers a valuable opportunity for timely interventions to enhance cognitive reserve, preserve cognitive function, and potentially prevent further deterioration. However, the considerable heterogeneity in cognitive trajectories among PD patients complicates prognostication and poses challenges for clinical trials aimed at addressing this critical aspect of the disease [18]. Therefore, predictive biomarkers for cognitive decline in PD are urgently needed to improve early detection and provide insights into the mechanisms driving cognitive decline in certain PD patients while sparing others [19]. Despite this urgency, practical methodologies integrating baseline demographics, clinical assessments, and blood biomarkers for early detection of PD-related cognitive decline remain limited. Addressing this critical gap, this study aims to provide a straightforward, reliable, and easy-to-use model for predicting cognitive decline in early PD using machine learning (ML) techniques and accessible baseline features.

Biomarkers play a pivotal role in PD, offering promise for early diagnosis, disease monitoring, and clinical trial design. Substantial evidence supports the idea that converting α-synuclein from soluble monomers to aggregated, insoluble forms in the brain is a hallmark of PD pathology [20]. This pathological process may also be found in human bodily fluids such as cerebrospinal fluid (CSF) and blood plasma [21,22]. Extensive research has delved into various biomarker types, encompassing clinical, genetic, CSF, and imaging biomarkers. These are increasingly pivotal in predicting cognitive decline during early diagnosis and disease prognostication [23,24,25,26,27]. Specifically, in CSF, higher levels of phosphorylated tau (p-tau) and lower levels of amyloid β42 have been linked to an elevated risk of dementia in PD patients [28,29]. Additionally, elevated neurofilament light chain (NfL) levels in CSF have been shown to predict cognitive decline, further underscoring the importance of these biomarkers in understanding and managing the disease [30].

While CSF biomarkers have shown significant promise, blood biomarkers stand out as particularly advantageous in PD due to their accessibility and cost-effectiveness compared to CSF and imaging biomarkers [31,32]. Prior research has explored the relationship between blood biomarkers and cognitive decline in early PD. For instance, increased physical activity has been shown to attenuate the vulnerability associated with the apolipoprotein E ε4 (APOE ε4) allele to early cognitive decline in patients with PD [33]. Another study highlighted the potential of elevated α-synuclein and total tau (t-tau), along with reduced amyloid-beta-40 (Aβ-40) levels, as biomarkers for the early detection of cognitive impairment in PD patients [34]. Additionally, a pilot study suggested that lower serum uric acid levels in the early stages of the disease may be associated with the later development of MCI [35]. Recent findings by Sekiya, et al. (2022) [36] further highlight the widespread presence of α-synuclein oligomers in various brain regions of PD patients, especially in the neocortex, and their association with cognitive impairment, suggesting their potential significance in early PD pathology. Furthermore, elevated plasma NfL levels and reduced epidermal growth factor levels have been linked to cognitive decline in PD patients [37,38,39]. Despite these advancements, studies investigating plasma biomarkers and correlations to cognitive decline in PD remain limited.

To the best of our knowledge, no existing risk prediction models have utilized ML methods incorporating baseline clinical, demographic, and blood biomarkers to predict the risk of cognitive decline in early PD. This study aims to fill this gap by developing a risk prediction model using ML algorithms capable of detecting complex patterns and interactions not discernible through traditional analysis methods.

Previous studies have explored various approaches to predicting cognitive decline in PD using different data sources and methodologies. For instance, a study used data from the Parkinson’s Progression Markers Initiative (PPMI) to accurately predict cognitive impairment at a 2-year follow-up [40]. Combining age, non-motor assessments, dopamine transporter (DAT) imaging, and CSF biomarkers effectively predicted Montreal Cognitive Assessment (MoCA) scores at the 2-year follow-up in newly diagnosed PD patients. Another study, also using PPMI data, developed a multimodal ML model to predict cognitive decline in early PD patients by utilizing the change in MoCA scores as the outcome, calculated from the difference between the baseline and 4-year follow-up data [41]. Additionally, a cross-sectional study using data from the Early Parkinson’s disease Longitudinal Singapore (PALS) study examined cognitive impairment by comparing PD-MCI patients and those with normal cognition (PD-NC). This study highlighted the significant associations between PD-MCI and several factors, including triglycerides (TG), apolipoprotein A1 (ApoA1), and the SNCA rs6826785 genetic marker, suggesting their potential role in early cognitive decline in PD patients [42].

Building on these insights, the current study utilized data from the PALS cohort to develop a risk prediction model for predicting cognitive decline over a five-year period in individuals with early PD by incorporating baseline characteristics and employing various ML algorithms.

## 2. Materials and Methods

### 2.1. Study Design and Population

This study utilized data from the PALS prospective cohort study to predict cognitive decline using ML techniques. Data were used from 214 PD patients, collected over five years between 2014 and 2018, with all participants meeting the National Institute of Neurological Disorders and Stroke (NINDS) clinical criteria for PD. Participants had to have more than 6 years of education and were able to read and write English or Mandarin to enroll in the study. Exclusion criteria included significant medical conditions hindering regular follow-up and orthopedic issues potentially affecting study outcomes. Functional status was measured using the Hoehn and Yahr (HY) rating scale, while motor symptom severity was assessed via the Movement Disorder Society-Unified Parkinson’s disease Rating Scale (MDS-UPDRS) Part III. Cognitive function was assessed using the MoCA. Where dopaminergic therapy had begun, the dosage was calculated and reported as cumulative levodopa equivalent daily dose (LEDD) [43,44].

Patients were defined as having ‘early PD’ based on the following inclusion criteria: (i) motor symptoms within two years, and (ii) diagnosis of PD within one year according to the NINDS criteria as determined by a specialist in movement disorders. Ethics approval was obtained from the Singapore Health Services Centralized Institutional Review Board (CIRB) for the use of human participants in this study, and all participants provided informed written consent. After excluding patients with missing MoCA scores for the first three years, 193 PD patients were included in the final analysis.

### 2.2. Outcome Definition

In the context of early PD, the MoCA is used as a key measure to assess cognitive decline. For the purposes of this study, cognitive decline was defined as either a one-unit annual decrease in MoCA score or a one-unit decline observed over two consecutive years during the five-year follow-up period. This threshold of a one-unit decline is clinically relevant, as even a small change can indicate an early sign of cognitive deterioration in early PD.

### 2.3. Input Variables

Input variables included baseline demographics, clinical assessments, and blood biomarkers. The baseline demographics included age, gender, years of education, body mass index (BMI), smoking status, alcohol consumption, coffee consumption, and tea consumption. Clinical assessments encompassed standing and lying systolic blood pressure (SBP), standing and lying diastolic blood pressure (DBP), HY, total MoCA score, total motor score, diabetes mellitus, hypertension, and hyperlipidemia. The blood biomarkers analyzed were suppression of tumorigenicity 2 (ST2), NfL, t-tau, phosphorylated tau at threonine 181 (p-tau181), apolipoprotein E (APOE), and alpha-synuclein gene promoter (REP1).

### 2.4. Data Imputation and Transformation

Missing values were imputed using a random forest-based imputation method, which estimates missing values by leveraging relationships observed in existing data [45]. This approach imputes missing data using mean/mode, and then iteratively fits a random forest (RF) to predict the missing values until a stopping criterion or maximum iterations are reached. Continuous input features were transformed into binary variables for easier clinical interpretation. For input variables without well-established cut-off points, including blood biomarkers and total motor score, the Youden index was used. This method, which incorporates both sensitivity and specificity, is a commonly used measure of overall diagnostic performance. It identifies the cut-off point that optimizes the biomarker’s differentiating ability when equal weight is assigned to both sensitivity and specificity [46].

### 2.5. Feature Selection

Initially, 24 variables were included in the study. The RF importance was calculated for each feature. The mean RF importance across all features was used as a threshold, and features with an RF importance below mean were excluded from the dataset.

### 2.6. Statistical Analysis and ML Methods

Descriptive statistics, including mean and standard deviation (SD) or median and first and third quartile, were reported for numeric variables, depending on the normality assumption, while categorical variables were presented as frequency and percentages. Univariate logistic regression analysis was performed to investigate the association of baseline patients’ characteristics with progression outcome, and odds ratios (OR) along with 95% confidence interval (CI) were calculated. Four ML methods including AutoScore, RF, k-nearest neighbors (KNN), and neural network (NN), along with logistic regression as a baseline statistical approach, were employed. To ensure optimal model performance, hyperparameter tuning was performed for all methods using 10-fold cross-validation. For RF, a grid search was employed to tune the number of trees (ntree), the number of variables selected at each split (mtry), the minimum node size (nodesize), and the maximum number of terminal nodes (maxnodes). The following values were explored: ntree = (100, 200, 500), mtry = (3, 4, 5), nodesize = (5, 10, 15), and maxnodes = (5, 10, 20). The optimal parameters were chosen based on cross-validated performance metrics, specifically the area under the curve (AUC). For NN, one hidden layer was considered, and hyperparameters including the number of hidden units (size = (1, 2, 3, 4, 5)) and weight decay (decay = (0, 0.01, 0.1)) were optimized, with accuracy as the performance metric. Similarly, for KNN, the number of neighbors was optimized within the range of 1 to 10 using grid search, also with accuracy as the performance metric. For AutoScore, as all variables were initially binary, a score table was generated from the model outputs to create interpretable clinical scores. Following hyperparameter tuning, models with the optimal parameters were compared using performance metrics including AUC, sensitivity, and specificity, along with their corresponding 95% CI. Sensitivity and specificity were determined by identifying the optimal threshold on the receiver operating characteristic (ROC) curve, defined as the point closest to the top-left corner, representing a balance between high sensitivity and specificity.

Each ML method was evaluated using the following two modeling strategies: (i) including all variables, and (ii) selecting the ten most important variables based on feature importance scores derived from the RF importance approach. AUC as an overall accuracy metric was used to identify the best-performing model. Model calibration was evaluated using a binned plot, which is recommended for smaller datasets. In this approach, predicted probabilities are grouped into 10 equal-sized bins, and for each bin, the midpoint of the predicted probability is plotted against the true fraction of positive cases. If the model is well calibrated, the points will fall near the diagonal line [47]. A risk score table for the model utilizing selected variables was generated using the AutoScore method, an easy-to-use ML algorithm designed to facilitate risk assessment [48]. Statistical significance was set at *p*-value < 0.05. All data analyses were conducted using R software 4.4.2. (R Core Team (2024); R: A Language and Environment for Statistical Computing. R Foundation for Statistical Computing, Vienna, Austria. https://www.R-project.org, accessed on 30 November 2024).

## 3. Results

### 3.1. Baseline Characteristics and Descriptive Statistics by Outcome

A total of 193 early PD patients completed baseline assessments and were included in our study, with 58% of the participants being male. At baseline, the mean age was 63.6 years (SD = 8.94 years), and the mean years of education was 10.7 years (SD = 4.37 years). The descriptive statistics of baseline variables are detailed in Table 1. Cognitive decline as the primary outcome was observed in 44 (23%) subjects. Significant findings include that patients with 10 or more years of education had a lower risk of cognitive decline compared to those with fewer years (OR = 0.36, *p*-value = 0.006). Elevated lying SBP, lying DBP, and standing SBP were associated with a higher risk of cognitive decline (OR = 2.13, *p*-value = 0.045; OR = 2.60, *p*-value = 0.009; OR = 2.13, *p*-value = 0.045, respectively).

### 3.2. Feature Selection Analysis

**Figure 1:**
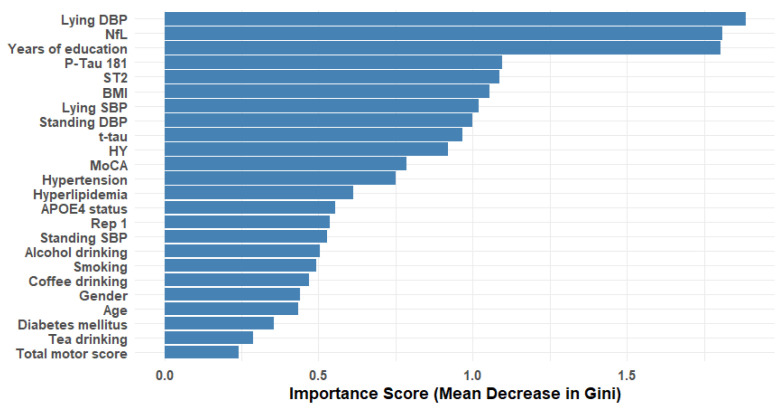
Feature importance ranked by mean decrease in Gini score.

RF importance scores were calculated for each feature (see Figure 1). The mean RF importance across all features was used as a threshold, and features with an RF importance score below the mean were excluded. This strategy selected 10 out of the initial 24 features: lying DBP, NfL, years of education, p-tau181, ST2, BMI, lying SBP, standing DBP, t-tau, and HY scale.

### 3.3. Model Performance and ROC Analysis

The results of the various ML methods are summarized in Table 2. In Model 1, which included all variables, the RF algorithm achieved the highest AUC at 0.999, indicating near-perfect discrimination between patients with and without cognitive decline. The NN also performed exceptionally well, with an AUC of 0.996. AutoScore and logistic regression showed moderate performance, with AUCs of 0.797 and 0.806, respectively. KNN had the lowest AUC at 0.766. In terms of sensitivity, both RF and NN were outstanding, with sensitivities of 1.000 and 0.977, respectively, highlighting their excellent ability to identify patients with cognitive decline. AutoScore had the lowest sensitivity at 0.636. In Model 2, which included the ten most important variables, RF again had the highest AUC at 0.930, followed by NN and KNN with AUCs of 0.918 and 0.843, respectively. Logistic regression and AutoScore exhibited similar moderate performance, with AUCs of 0.770 and 0.771, respectively. NN achieved the highest sensitivity at 0.841, indicating strong detection capability with fewer variables. AutoScore, RF, and KNN all performed well, each with a sensitivity of 0.818. Overall, RF consistently showed the highest AUC across both models, demonstrating superior performance in distinguishing between patients with and without cognitive decline. NN also performed strongly, particularly in Model 2, where it exhibited the highest sensitivity.

### 3.4. Calibration of the Predictive Models

**Figure 2:**
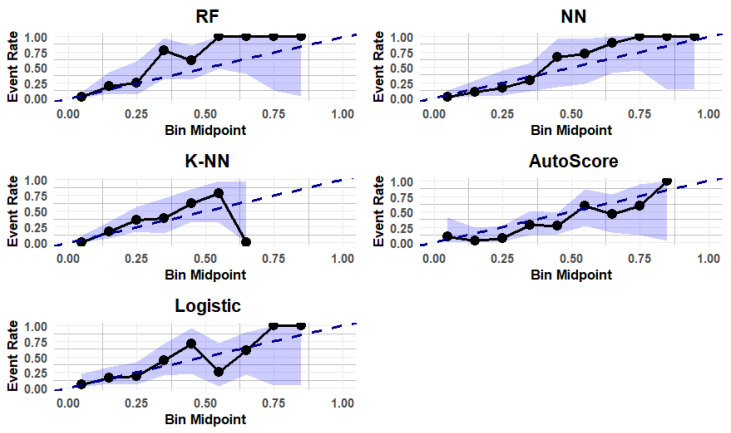
Calibration plot with 99% CI for Model 2 across different methods. The black points indicate the observed event rates for each bin, while the black line connects these points to show the trend. The dashed dark blue diagonal line represents the ideal calibration line, indicating perfect agreement between predicted probabilities and observed event rates.

Calibration was assessed using a binned plot for both models. Figure 2 presents the calibration results specifically for Model 2, featuring a binned plot with a 99% CI based on the internal data. The plot shows that both RF and NN tend to overestimate the predicted probabilities, while KNN underestimates them. In contrast, logistic regression and AutoScore demonstrate better calibration performance.

### 3.5. Score of Risk Factors Based on AutoScore Algorithm

The risk score generated for model 2 by AutoScore algorithm is summarized in Table 3. The high sensitivity demonstrated by AutoScore in this model highlights the importance of the identified risk factors. Results indicate that lower education (<10 years), higher NfL (≥21.5 pg/mL), higher ST2 (≥14,185.8 pg/mL), higher t-tau (≥2.1 pg/mL), higher p-tau181 (≥27.1 pg/mL), higher standing and lying DBP (≥80 mmHg), higher lying SBP (≥140 mmHg), higher BMI (≥25 kg), and higher HY scale (≥2) are significant risk factors that increase the likelihood of cognitive decline in early PD patients.

### 3.6. Summary of Key Findings

Overall, feature selection using RF importance scores narrowed down the variables to ten, which were used in various ML methods. The RF algorithm demonstrated the highest AUC (0.999) in Model 1, and also achieved the highest AUC (0.930) in Model 2, which incorporated the most important variables. Both RF and NN exhibited high sensitivity, particularly in identifying patients with cognitive decline. The risk factors identified through AutoScore emphasize the clinical relevance of these variables in predicting cognitive decline in early PD patients.

## 4. Discussion

Cognitive impairment is one of the most common non-motor symptoms in PD and can be more devastating for both patients and caregivers than motor symptoms [49]. Cognitive decline is increasingly recognized as a prevalent issue even in newly diagnosed PD patients [8,10]. The search for objective biomarkers is driven by their potential to enhance early and accurate diagnosis, monitor disease progression, and optimize clinical trial design and interpretation. While alpha-synuclein remains a promising biomarker candidate, the complex and heterogeneous nature of PD underscores the necessity for a comprehensive biomarker panel [31,32]. In light of these considerations, this study aimed to develop accurate predictive models for cognitive decline in early PD patients. Leveraging a combination of blood biomarkers, clinical data, and demographic characteristics, ML techniques were employed to achieve this objective.

This study compared two different modeling strategies to predict cognitive decline in early PD patients, emphasizing the importance of practicality in clinical settings. Model 1, which included all available variables, demonstrated the highest performance metrics, particularly with the RF and NN algorithms. However, the complexity and cost associated with obtaining comprehensive datasets limit its utility in routine clinical practice. In contrast, Model 2, incorporating only the ten most significant variables identified through feature selection, not only maintained strong performance metrics but also enhanced practicality for clinicians. Notably, this model showcased remarkable sensitivity with the NN algorithm, suggesting its potential to effectively detect cognitive decline using a streamlined approach. By focusing on easily obtainable variables, such as years of education and blood pressure, Model 2 could facilitate timely interventions, allowing healthcare providers to identify patients at risk for rapid cognitive decline. Implementing such practical models in clinical workflows could significantly improve early detection and management strategies, ultimately enhancing patient outcomes in early-stage PD.

The ten primary variables derived from feature selection, comprising a blend of demographics, clinical parameters, and blood biomarkers, included years of education, BMI, NfL, t-tau, p-tau181, ST2, standing DBP, lying DBP, lying SBP, and HY scale. Notably, several of these features have been identified as significant risk factors in prior research, with further details provided below.

In this study, BMI emerged as a significant demographic variable with potential implications for managing early PD. Although research specifically examining the effect of BMI on early PD is lacking, several studies have explored its association with cognitive decline in PD. For instance, an analysis of data from PPMI identified that higher baseline BMI, along with modifiable comorbidities such as depression and sleep disorders, contributed to an accelerated rate of cognitive decline in PD patients [50]. Similarly, another study using PPMI data found that PD patients with a metabolically unhealthy normal weight (MUNW) phenotype experienced more rapid cognitive decline, particularly in global cognition and visuospatial perception, over a 48-month period compared to those in other BMI-metabolic status categories [51]. Conversely, Yoo et al. (2019) reported that PD patients with a higher-than-normal BMI at diagnosis exhibited a slower cognitive decline and a reduced risk of developing dementia over a six-year period compared to those with under/normal weight, suggesting that a higher BMI may have a protective effect against cognitive deterioration in PD [52]. Additionally, Kim et al. (2012) observed that a decrease in BMI during the initial six months of follow-up in PD patients could serve as an early indicator of future dementia risk, enabling clinicians to predict a faster rate of cognitive decline [53]. These findings underscore the importance of monitoring BMI in PD patients, as it may inform clinical decisions regarding interventions aimed at preserving cognitive function and improving overall patient outcomes.

In addition to BMI, years of education also emerged as a significant demographic predictor in this study. This finding is consistent with a recent cross-sectional study using PALS data, which demonstrated that fewer years of education are associated with higher MDS-UPDRS Part III and an elevated risk of MCI in early PD [42]. Lower educational attainment may therefore be a marker for greater vulnerability to motor and cognitive declines in PD, underscoring the potential role of education in influencing disease progression and patient outcomes.

In the present study, standing DBP, lying DBP, and lying SBP emerged as significant clinical predictors of cognitive decline in early PD. This aligns with previous research underscoring the role of hypertension in cognitive decline among early PD patients. Previous research has shown that PD-MCI patients exhibited significantly higher diastolic blood pressure variability (BPV) during follow-up compared to those with non-MCI PD, suggesting BPV as a potential predictive marker of cognitive decline [54]. Additionally, an analysis using PPMI data indicated that elevated visit-to-visit variability in systolic blood pressure (systolic VIM) was associated with a faster decline in global cognitive function, assessed by the MoCA score, in PD-MCI patients [55]. Further emphasizing the importance of blood pressure management, another study found that, on average, every 10 mmHg increase in pulse pressure was associated with a 0.08 reduction in cognitive Z-scores in early PD [56]. These findings collectively highlight the critical need for effective blood pressure management in early PD to mitigate the risk of cognitive decline.

Through this study, the HY scale emerged as a significant predictor of cognitive decline in early PD, underscoring its clinical relevance beyond motor symptom assessment. This finding aligns with previous research demonstrating a strong association between motor impairment severity, as measured by the HY scale, and cognitive deficits in PD patients. For example, a study by Siciliano et al. (2017) compared cognitive performance in de novo PD patients and found that those at HY stage II scored significantly lower on neuropsychological tests compared to those at HY stage I, indicating that greater motor impairment correlates with increased cognitive dysfunction [57]. Additionally, the predictive power of the HY scale for disease progression is further supported by studies such as the PASADENA trial, a Phase II randomized, double-blind, placebo-controlled study investigating the efficacy and safety of prasinezumab in early PD [58], and analyses using PPMI data [59]. These studies identified the HY stage, along with other biomarkers like dopamine transporter SPECT imaging, as the key predictors of clinical progression in early PD. These findings highlight the critical role of the HY scale in the early detection and management of PD, aiding clinicians in predicting and potentially mitigating cognitive decline.

Regarding blood biomarkers, four biomarkers, NfL, p-tau181, t-tau, and ST2, were identified as significant predictors of cognitive decline in early PD. Our findings for NfL align closely with previous research, reinforcing its role as a valuable prognostic biomarker. For instance, one study demonstrated that elevated serum NfL levels are positively associated with an increased risk of early PD-related symptoms, suggesting that serum NfL could serve as a promising biomarker for early PD [60]. Additionally, another study discovered, through a study using PALS data, that higher plasma NfL levels were linked to a frontal pattern of neurodegeneration, which also correlated with cognitive performance in early PD [61]. This supports the potential future role of plasma NfL as an accessible biomarker for neurodegeneration and cognitive dysfunction in PD. Ng et al. (2020) further highlighted that higher plasma NfL levels were associated with worse cognition and motor function in the postural instability gait disorder (PIGD) subtype of PD, predicting motor and cognitive decline over two years [62]. Similarly, Aamodt et al. (2021) reported that PD participants with high plasma NfL levels were significantly more likely to develop incident cognitive impairment (HR = 5.34, *p*-value = 0.005). Although their ROC analysis demonstrated only modest performance for plasma NfL alone in predicting the conversion from normal cognition to MCI or dementia, they noted that incorporating plasma NfL into a multi-marker panel could enhance predictive accuracy [37]. In line with these findings, Batzu et al. (2022) reported that higher plasma NfL levels in PD patients were associated with lower Mini-Mental State Examination (MMSE) scores at baseline, even after adjusting for age, gender, and education [63].

To our knowledge, there have been no extensive studies specifically exploring the roles of blood biomarkers including t-tau, p-tau181, and ST2 in predicting cognitive decline in early PD. In this context, Batzu et al. (2022) conducted a cross-sectional study that found significantly higher plasma p-tau181 concentrations in PD subjects compared to healthy controls at baseline [63]. However, their follow-up over two years did not reveal a significant association between plasma p-tau181 levels and either baseline or longitudinal cognitive performance. Another study highlighted the potential of elevated α-synuclein and t-tau, along with reduced Aβ-40 levels, as biomarkers for the early detection of cognitive impairment in PD patients [34].

Most research in this area has focused on CSF biomarkers. For instance, Almgren et al. (2023) used PPMI data to develop a ML model for predicting cognitive decline in de novo PD, incorporating CSF biomarkers, clinical test scores, basic demographics, and baseline cognition [41]. Their findings showed that higher levels of CSF beta-amyloid were significantly associated with less cognitive decline, while higher baseline MoCA scores, elevated CSF t-tau, anxiety, and autonomic dysfunction were linked to greater cognitive decline. Similarly, Tao et al. (2022) investigated the associations between non-motor symptoms and CSF biomarkers in early PD using PPMI data [64]. They found that PD patients with cognitive impairment had significantly lower levels of CSF α-synuclein, Aβ_1–42_, and t-tau compared to PD patients without cognitive impairment. Additionally, Terrelonge et al. (2016) explored the role of CSF biomarkers in predicting cognitive impairment in early PD, revealing that lower baseline levels of CSF Aβ_1–42_ were significantly associated with a higher risk of cognitive impairment over a two-year period, while no significant associations were found for t-tau or p-tau181 [65]. These findings emphasize the importance of CSF biomarkers as early indicators of cognitive decline risk in PD, underscoring their potential clinical utility for early diagnosis and targeted intervention in PD-related cognitive impairment.

In terms of ST2, a study measuring the plasma soluble decoy receptor form of ST2 (sST2) levels in controls and patients with Alzheimer’s disease (AD), frontotemporal dementia (FTD), and PD found that sST2 levels were elevated across all disease groups compared to controls, with the highest levels observed in FTD, followed by AD and PD [66,67]. However, to our knowledge, no studies have specifically investigated plasma ST2 levels in the context of early-stage PD. This highlights the novelty of our study in exploring the association of ST2 with cognitive decline in early PD.

In other words, this study is among the first to explore the association of plasma biomarkers, including t-tau, p-tau181, and ST2, with cognitive decline in early PD. This novel approach provides new insights into how these plasma biomarkers might predict cognitive deterioration in early-stage PD.

The performance of different ML methods in predicting cognitive decline in early PD was evaluated, with RF and NN consistently showing superior results compared to AutoScore and KNN. Model 1, which included all available variables, demonstrated the highest performance, while Model 2, focusing on the top ten variables, provided a more practical approach with notable performance in predicting cognitive decline in early PD.

Previous studies have leveraged ML algorithms to enhance the prediction of cognitive decline and other outcomes in PD. For instance, Zhang et al. (2023) used demographic variables, hospital admission data, and clinical assessments, while grouping predictors based on their cost and accessibility, to build models predicting PD risk. Penalized logistic regression and XGBoost emerged as the most accurate algorithms, with penalized logistic regression achieving an AUC of 0.94 [3]. Deng et al. (2023) conducted a cross-sectional study on PALS data, identifying eight key variables associated with MCI in early PD using ShapleyVIC-assisted and backward selection methods [42]. Their final model included fewer years of education, a shorter history of hypertension, higher MDS-UPDRS motor scores, elevated levels of TG and ApoA1, and noncarrier status of the SNCA rs6826785 genetic marker. These findings align with the present study, which also identified fewer years of education and a history of hypertension as significant predictors of cognitive decline. The combined insights from these studies underscore the importance of a multifaceted approach in using ML to predict cognitive outcomes as a longitudinal outcome in early PD, integrating demographic, clinical, biochemical, and genetic factors for more accurate and practical predictive models.

### Limitations and Future Avenues

This study employed 10-fold cross-validation to assess model performance; however, several limitations should be noted. The high AUC observed could be influenced by the small sample size, which may lead to overly optimistic performance estimates, even with 10-fold cross-validation. Although one-tenth of the data are set aside for validation in each iteration, small datasets can result in higher variance in performance metrics, and the results may not generalize well to larger, independent datasets. This raises concerns about producing biased performance estimates. Therefore, a more conservative approach, such as nested cross-validation, may provide more reliable performance estimates. Additionally, the calibration results for Model 2 showed that while RF and NN tend to overestimate predicted probabilities, KNN underestimates them. In contrast, logistic regression and AutoScore exhibit better calibration performance. However, these results should be interpreted with caution due to the small sample size and the use of the same dataset for both training and evaluation. These limitations highlight the necessity for further validation with independent, external datasets to ensure the robustness and generalizability of the findings.

## 5. Conclusions

This study demonstrates the potential of ML methods in accurately predicting cognitive decline in individuals with early-stage PD. By integrating baseline demographic, clinical, and blood biomarker data, these models offer valuable insights for the early identification of patients at a high risk of cognitive deterioration, providing opportunities for timely interventions and improved patient outcomes. While a comprehensive model incorporating all available variables achieved the highest predictive performance, the practicality of utilizing certain biomarkers in clinical settings may be limited due to their cost and accessibility. A more streamlined model focusing on key biomarkers, however, maintained strong predictive capabilities, offering a more practical and feasible approach for real-world clinical implementation. These findings underscore the theoretical implications of integrating data-driven approaches in neurodegenerative disease management and highlight opportunities for translating ML models into clinical practice. Future research should explore strategies to enhance model interpretability, validate findings across diverse populations, and assess long-term impacts on patient care. Additionally, further methodological developments are needed to optimize biomarker selection and address practical implementation challenges, paving the way for broader adoption of predictive analytics in personalized medicine.

## Figures and Tables

**Table 1:** Summary statistics of demographic, clinical assessments, blood biomarkers, and their associations with progression outcomes using univariate logistic regression.

	Total	No Progression	Progression	OR (95% CIs)	*p*-Value
	N = 193	N = 149	N = 44		
Demographic Characteristics					
Male Gender	112 (58.0%)	85 (57.0%)	27 (61.4%)	1.2 (0.6, 2.4)	0.737
Smoker	56 (29.0%)	43 (28.9%)	13 (29.5%)	1.0 (0.5, 2.1)	1.000
Years of education (≥10 years)	135 (69.9%)	112 (75.2%)	23 (52.3%)	0.4 (0.2, 0.7)	0.006
Tea drinking	180 (93.3%)	138 (92.6%)	42 (95.5%)	1.6 (0.4, 11.5)	0.736
Coffee drinking	175 (90.7%)	135 (90.6%)	40 (90.9%)	1.0 (0.3, 3.8)	1.000
Alcohol drinking	125 (64.8%)	101 (67.8%)	24 (54.5%)	0.6 (0.3, 1.1)	0.151
BMI (>25 kg/m^2^)	64 (33.2%)	48 (32.2%)	16 (36.4%)	1.2 (0.6, 2.4)	0.740
Age (>65 years)	97 (50.3%)	72 (48.3%)	25 (56.8%)	1.4 (0.7, 2.8)	0.413
**Clinical Assessments**					
Lying SBP (≥140 mmHg)	95 (49.2%)	67 (45.0%)	28 (63.6%)	2.1 (1.1, 4.3)	0.045
Lying DBP (≥80 mmHg)	67 (34.7%)	44 (29.5%)	23 (52.3%)	2.6 (1.3, 5.2)	0.009
Standing SBP (≥140 mmHg)	85 (44.0%)	63 (42.3%)	22 (50.0%)	1.4 (0.7, 2.7)	0.463
Standing DBP (≥80 mmHg)	95 (49.2%)	67 (45.0%)	28 (63.6%)	2.1 (1.1, 4.3)	0.045
Diabetes mellitus	31 (16.1%)	25 (16.8%)	6 (13.6%)	0.8 (0.3, 2.0)	0.791
Hypertension	88 (45.6%)	68 (45.6%)	20 (45.5%)	1.0 (0.5, 2.0)	1.000
Hyperlipidemia	92 (47.7%)	73 (49.0%)	19 (43.2%)	0.8 (0.4, 1.6)	0.613
MoCA	26 [23.0, 28.0]	26 [23.0, 28.0]	26 [23.0, 28.0]	1.0 (0.9, 1.1)	0.771
Total motor score	20.0 [15.0; 26.0]	19.0 [15.0; 26.0]	22.0 [17.0; 29.0]	1.0 (1.0, 1.1)	0.062
HY	2.00 [1.0; 3.0]	2.00 [1.50; 2.0]	2.00 [2.00; 2.0]	2.0 (0.8, 4.8)	0.112
**Blood Biomarkers**					
APOE4 (Non-carriers)	153 (79.3%)	120 (80.5%)	33 (75.0%)	0.7 (0.3, 1.7)	0.559
REP1 (Short)	88 (45.6%)	66 (44.3%)	22 (50.0%)	1.3 (0.6, 2.5)	0.620
ST2	11,600 [8750; 14,800]	11,500 [8400; 14,900]	12,600 [9430; 14,800]	1.0 (1.0, 1.0)	0.375
NfL	13.7 [10.1; 18.9]	13.9 [10.2; 18.7]	13.3 [9.9; 21.7]	1.0 (1.0, 1.1)	0.702
t-tau	1.17 [0.9; 1.5]	1.1 [0.9; 1.6]	1.3 [0.9; 1.5]	1.3 (0.9, 1.8)	0.350
p-tau181	20.3 [15.7; 24.8]	20.50 [15.4; 24.3]	20.1 [15.8; 28.9]	1.0 (1.0, 1.1)	0.666

Data are expressed as frequency (%) or median (quartile); *p*-values are from univariate logistic regression models assessing the association of each variable with cognitive decline progression. Abbreviations: N: number, OR: odds ratio, CIs: confidence intervals, BMI: body mass index, SBP: systolic blood pressure, DBP: diastolic blood pressure, MoCA: Montreal Cognitive Assessment, HY: Hoehn and Yahr scale, APOE: apolipoprotein E, REP1: alpha-synuclein gene promoter, ST2: suppression of tumorigenicity 2, NfL: neurofilament light chain, t-tau: total tau, p-tau181: phosphorylated tau at threonine 181.

**Table 2:** The performance of four ML methods * under two modeling strategies.

Algorithm	AUC (95% CI)	Sensitivity (95% CI)	Specificity (95% CI)
Model 1: All Variables			
AutoScore	0.797 (0.720, 0.8736)	0.636 (0.500, 0.773)	0.825 (0.765, 0.879)
RF	0.999 (0.997, 1.000)	1.000 (0.920, 1.000)	0.987 (0.952, 0.998)
KNN	0.766 (0.690, 0.842)	0.750 (0.597, 0.868)	0.678 (0.596, 0.752)
NN	0.996 (0.989, 1.000)	0.977 (0.880, 0.999)	0.987 (0.952, 0.998)
Logistic	0.806 (0.731,0.881)	0.682 (0.524, 0.814)	0.819 (0.747, 0.877)
**Model 2: Top Ten Variables**			
AutoScore	0.771 (0.691,0.851)	0.818 (0.705, 0.909)	0.631(0.557, 0.705)
RF	0.930 (0.889,0.971)	0.818 (0.673, 0.918)	0.872 (0.808, 0.921)
KNN	0.843 (0.788,0.899)	0.818 (0.673, 0.918)	0.711 (0.632, 0.783)
NN	0.918 (0.872,0.965)	0.841 (0.699, 0.934)	0.832 (0.762, 0.888)
Logistic	0.770 (0.690, 0.849)	0.795 (0.647, 0.902)	0.631 (0.548, 0.708)

* Results obtained through ROC analysis on the training dataset using 10-fold cross-validation. Top ten variables: lying DBP, NfL, years of education, p-tau 181, ST2, BMI, lying SBP, standing DBP, t-tau, and HY.

**Table 3:** Risk scores generated by AutoScore algorithm * for Model 2.

Variable	Interval	Partial Score
Lying DBP	Normal	0
	High	16
NfL	Normal	0
	High	18
Years of education	≥10	0
	<10	16
p-tau 181	Normal	0
	High	11
ST2	Normal	0
	High	9
BMI	<25	0
	≥25	3
Lying SBP	Normal	0
	High	1
Standing DBP	Normal	0
	High	11
t-tau	Normal	0
	High	11
HY	<2	0
	≥2	4

* AutoScore, an interpretable ML-based tool for generating automatic clinical score, provides risk factor scores for each feature. This capability translates complex model predictions into a more understandable format for clinical decision making. Abbreviations: DBP: diastolic blood pressure, NfL: neurofilament light chain, p-tau 181: phosphorylated tau at threonine 181, ST2: suppression of tumorigenicity 2, BMI: Body mass index, SBP: systolic blood pressure, t-tau: total tau, HY: Hoehn and Yahr scale.

## Data Availability

The study data will be made available upon reasonable request to the corresponding author. The data are not publicly available due to privacy and ethical concerns.

## Abbreviations

PD	Parkinson’s disease
CD	Cognitive decline
MoCA	Montreal Cognitive Assessment
AUC	Area under the curve
MCI	Mild cognitive impairment
PDD	Parkinson’s disease dementia
ML	Machine learning
RF	Random forest
CSF	Cerebrospinal fluid
PPMI	Parkinson’s Progression Markers Initiative
ApoA1	Apolipoprotein A1
TG	Triglycerides
HY	Hoehn and Yahr
MDS-UPDRS	Movement Disorder Society-Unified Parkinson’s disease Rating Scale
BMI	Body mass index
SBP	Systolic blood pressure
DBP	Diastolic blood pressure
ST2	Suppression of tumorigenicity 2
NfL	Neurofilament light chain
t-tau	Total tau
p-tau 181	Phosphorylated tau at threonine 181
APOE	Apolipoprotein E
REP1	Alpha-synuclein gene promoter
OR	Odds ratio
SD	Standard deviation
CI	Confidence interval
KNN	K-nearest neighbors
NN	Neural network
ROC	Receiver operating characteristic
AD	Alzheimer’s disease
FTD	Frontotemporal dementia
